# Nutrient Content and Nutritional Water Productivity of Selected Grain Legumes in Response to Production Environment

**DOI:** 10.3390/ijerph14111300

**Published:** 2017-10-26

**Authors:** Tendai Polite Chibarabada, Albert Thembinkosi Modi, Tafadzwanashe Mabhaudhi

**Affiliations:** Crop Science, School of Agricultural, Earth and Environmental Sciences, College of Agriculture, Engineering and Science, University of KwaZulu-Natal, Private Bag X01, Scottsville 3201 Pietermaritzburg, South Africa; modiAT@ukzn.ac.za (A.T.M.); mabhaudhi@ukzn.ac.za (T.M.)

**Keywords:** groundnut, dry bean, cowpea, bambara groundnut, groundnut, food and nutrition insecurity, yield, evapotranspiration

## Abstract

There is a need to incorporate nutrition into aspects of crop and water productivity to tackle food and nutrition insecurity (FNS). The study determined the nutritional water productivity (NWP) of selected major (groundnut, dry bean) and indigenous (bambara groundnut and cowpea) grain legumes in response to water regimes and environments. Field trials were conducted during 2015/16 and 2016/17 at three sites in KwaZulu-Natal, South Africa (Ukulinga, Fountainhill and Umbumbulu). Yield and evapotranspiration (ET) data were collected. Grain was analysed for protein, fat, Ca, Fe and Zn nutrient content (NC). Yield, ET and NC were then used to compute NWP. Overall, the major legumes performed better than the indigenous grain legumes. Groundnut had the highest NWP_fat_. Groundnut and dry bean had the highest NWP_protein_. For NWP_Fe, Zn and Ca_, dry bean and cowpea were more productive. Yield instability caused fluctuations in NWP. Water treatments were not significant (*p* > 0.05). While there is scope to improve NWP under rainfed conditions, a lack of crop improvement currently limits the potential of indigenous grain legumes. This provides an initial insight on the nutrient content and NWP of a limited number of selected grain legumes in response to the production environment. There is a need for follow-up research to include cowpea data. Future studies should provide more experimental data and explore effects of additional factors such as management practices (fertiliser levels and plant density), climate and edaphic factors on nutrient content and NWP of crops.

## 1. Introduction

Two billion people suffer from micronutrient deficiency, with nearly one billion being calorie deficient [1]. There is a gap between food supply and nutritional requirements, which has been attributed to a lack of nutritional considerations in crop production [2]. There is a need for a paradigm shift in current food production to consider nutrition outcomes [3]. Increasing food production and productivity should be tied to increasing nutrient density. In this regard, agriculture could simultaneously address the challenge of increasing food production and improving nutrition under limited resource availability. However, there are often challenges to linking disciplines as there are often no appropriate metrics for evaluating such linkages. In the case of quantifying the water-food-nutrition nexus, nutritional water productivity (NWP) has been proposed as a useful metric [4].

Nutritional water productivity is a measure of yield and nutrition outcome per unit of water consumed and would be applicable for sustainable food production given the limited water resources and modified diets [4,5] .To date, increasing food production under water scarcity has been evaluated using different metrics such as “water use efficiency” and “water productivity” [6,7,8,9,10]. On the other hand, nutritionists have quantified nutritional content of different foodstuffs and suggested diets for improving nutritional status of people. These efforts have been parallel and needed to be merged to address the challenge of producing more nutritious food under water scarcity. Nutritional water productivity would be a useful metric in the semi- and arid tropics (South Asia and sub-Saharan Africa) where water scarcity and food and nutrition insecurity are prevalent [3].

The high prevalence of food and nutrition insecurity has been attributed to dominance of starch in diets leading to poor dietary diversity. Diets lack in protein, micro nutrients and minerals [11,12,13,14]. This leads to various forms of malnutrition, including but not limited to, stunting, wasting and underweight in children under five, anaemia in women of the reproductive age, obesity and type 2 diabetes [1]. Dietary diversity has been recommended to alleviate malnutrition. Dietary diversity is defined as the number of different foods or food groups consumed over a given reference period [15]. Increasing the variety of foods across and within food groups ensures adequate intake of essential nutrients to promote good health. Grain legumes are being promoted in the semi- and arid tropics, as part of dietary diversity efforts. They are rich in proteins and some micronutrients [16,17,18], hence have the potential to alleviate malnutrition. The nutritional properties of grain legumes have been associated with reduction of environmental enteric dysfunction (EED) [19]—an incompletely defined syndrome of inflammation, reduced absorptive capacity, and reduced barrier function in the small intestine which is common among the rural poor in the semi- and arid tropics [20]. Crop diversification through inclusion of indigenous grain legumes in food and nutrition agendas has been proposed by several authors [3,21,22,23]. A study on nutrient content and NWP of indigenous and exotic vegetables observed that crops differed in their nutrient content and NWP [24]. For some micro nutrients, indigenous vegetables were more nutrient dense compared to the reference exotic vegetable swiss chard (*Beta vulgaris*).

In the semi- and arid tropics, water is one of the main limiting factors in agriculture. Yield of grain legumes has been observed to decrease with decreasing water availability [25,26,27]. Grain legumes have also been associated with yield instability across environments. There is not much information on how water availability and different environments affect nutritional content of grain legumes. Moreover, there is need to link yield, water use and nutritional content of grain legumes to establish the best yielding crops that use less water and are nutritionally dense. This should include indigenous grain legumes as they form part of crop diversification efforts. This information will be useful for promotion of grain legumes across different environments. It is hypothesised that nutrient content and NWP of crops will not vary with varying water availability and across environments. The aim of the study was therefore to determine the effect of production environment on NWP of selected indigenous and major grain legumes that share the same ecological niche and are usually consumed as whole grains by the rural population. The specific objectives were to determine nutrient content and NWP of selected indigenous [bambara groundnut (*Vigna subterranea*) and cowpea (*Vigna unguiculata*)] and major grain legumes [groundnut (*Arachis hypogaea*) and dry bean (*Phaseolus vulgaris*)] in response to (i) water regimes and (ii) environments.

## 2. Materials and Methods

### 2.1. Plant Material

**Figure 1:**
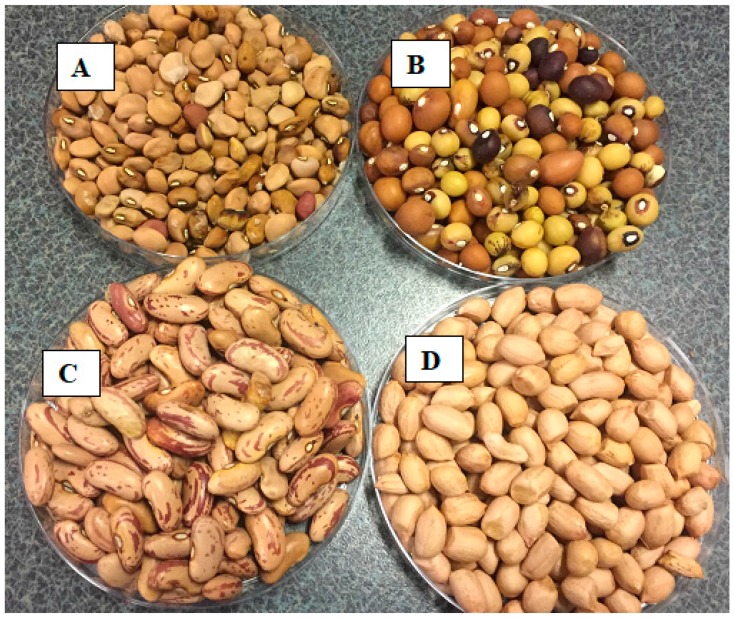
Seeds of selected varieties of indigenous grain legumes (**A** = cowpea—mixed brown; **B** = bambara groundnut—landrace) and major grain legumes (**C** = dry bean—Ukulinga; **D** = groundnuts—Kwarts).

Two major grain legumes that are recognised internationally (groundnut and dry bean) and two African indigenous grain legumes that are being promoted as healthy alternatives (bambara groundnut and cowpea) were selected for the study (Figure 1). Groundnut has high oil content and is usually consumed as a snack or processed to peanut butter or groundnut oil. Bambara groundnut, cowpea and dry bean, are normally harvested as dry grain and consumed after boiling them. Bambara groundnut and groundnut, form pods below ground while dry bean and cowpea form pods above ground. For the study, popular South African varieties of groundnut (Kwarts), dry bean (Ukulinga) and cowpea (mixed brown) were used for the study. For bambara groundnut, a mixed colour landrace from Jozini, South Africa was used. Kwarts is a variety suitable for warm dry areas [28]. Ukulinga is a high yielding variety of dry bean that is well adapted to most dry bean producing areas [29]. Mixed brown is a drought tolerant variety that is well adapted to most soils [30]. There was no information on the bambara groundnut landrace.

### 2.2. Site Description

Three sites (one on-station and two on-farm) were selected from KwaZulu-Natal Province, South Africa (Table 1). Ukulinga, which was the on-station farm, is a Research Farm, belonging to the University of KwaZulu-Natal. Ukulinga has access to irrigation. Umbumbulu and Fountanhill were on farm trials and did not have access to irrigation. Umbumbulu is a rural district in the eThekwini district of KwaZulu-Natal. Fountainhill is an Estate 2 km outside of Wartburg, KwaZulu-Natal.

### 2.3. Experimental Design and Trial Management

The experimental design at Ukulinga Research Farm, where there was access to irrigation, was a split-plot design arranged in randomised complete blocks with three replications. The main plots were irrigation regimes (optimum irrigation, deficit irrigation and rainfed) while the subplots were the grain legume crops (dry bean, groundnut and bambara groundnut). Irrigation scheduling in the optimum irrigation was based on 80% management allowable depletion (MAD), where water was maintained above 80% of total available water (TAW). The DI treatment was irrigated (MAD: 80% TAW) at the most sensitive to water stress growth stages (flowering and pod-filling stages). To determine the effect of environment, an experiment was conducted at the three sites (Fountainhill Estate, Ukulinga Research Farm and Umbumbulu Rural District) under rainfed conditions. At all sites, the experimental design was a randomised complete block design with three replications. There was no cowpea at Ukulinga. At Umbumbulu, trials only established during the 2016/17 season.

At all the sites, plot size (sub-plot at Ukulinga) was 18.75 m^2^. Plant population was 26,667 plants hectare^−1^ for cowpea, 66,667 plants hectare^−1^ for bambara groundnut and 88,889 plants hectare^−1^ for dry bean and groundnut. During 2015/16, trials were planted on 17 November 2015 at Ukulinga and 4 December 2015 at Fountainhill. During 2016/17, trials were planted on 30 November, 14 December and 16 January 2016 at Umbumbulu, Fountainhill and Ukulinga, respectively. At planting, a slow release organic fertiliser [Gromor accelerator (0.3% N, 0.15% P and 0.15% K)] was applied at a rate of 4000 kg·ha^−1^ using the band placement method. Rate of fertilizer application was based on results of fertility analysis conducted prior to the experiment. Results showed that to meet the nutrient requirements of the grain legumes under study, there was need to add 120 and 50 kg·ha^−1^ of N and P at Ukulinga and Fountainhill, while at Umbumbulu deficient N, P and K was 120, 50 and 10 kg·ha^−1^, respectively. For the duration of the trials, recommended best management practices (weeding, ridging and pest and disease control) for each crop were applied.

### 2.4. Measurements

#### 2.4.1. Yield and Yield Components

At harvest, six representative plants were randomly selected from each plot. Thereafter, the plants were air dried in a controlled environment situated at the UKZN Phytosanitary Unit until there was no change in total biomass. Pods were dehulled and grain mass was determined.

#### 2.4.2. Determination of Evapotranspiration (ET)

Evapotranspiration for each treatment was calculated as the residual of a soil water balance [31]:

ET = P + I − D − R − ΔSWC(1)

where ET = evapotranspiration (mm), P = precipitation (mm), I = irrigation (mm), D = drainage (mm), R = runoff (mm), and ΔSWC = changes in soil water content (mm).

Daily rainfall (mm) was obtained from weather stations within a 10 km radius from the sites. At Fountainhill and Umbumbulu, daily rainfall data was obtained from the South African Sugar Association (SASA) weather web portal (http://portal.sasa.org.za/weatherweb). At Ukulinga, daily rainfall data was obtained from an automatic weather station (AWS), which is part of the Agricultural Research Council – Institute for Soil, Climate and Water (ARC-ISCW) network of automatic weather stations. Changes in soil water content (SWC) were measured using a PR2/6 profile probe connected to an HH2 handheld moisture meter (Delta-T, Burwell, UK). The sensors of the PR2/6 profile probe are positioned to measure volumetric water content at six depths (0.10, 0.20, 0.30, 0.40, 0.60 and 1.00 m along the probe). The effective depth at Ukulinga was 0.40 m, hence the sensors positioned at 0.60 and 1.00 m were considered during analyses.

Drainage was considered as negligible. At Ukulinga, there was an impeding layer at 0.4 m which restricted downward movement of water beyond the root zone. At Fountainhill and Umbumbulu, drainage was considered negligible based on Dancette and Hall [32] where in semi- and arid environments drainage is negligible if the profile is not periodically saturated to drain excess water. Runoff (R) was not quantified during the trials. However, to account for its effect the United States Department of Agriculture–Soil Conservation Service (USDA-SCS) procedure was used to estimate the monthly effective rainfall that is stored in the root zone after subtracting the amount of rainfall lost to runoff [33]. The soil water balance was therefore simplified to;

ET= ER + I − ΔSWC(2)

where: ET = evapotranspiration = water use (mm), ER = effective rainfall (mm), I = irrigation (mm), and ΔSWC = changes in soil water content (mm). Values of ET in mm (depth) were then converted to m^3^ (volume) using the formula;

Volume (m^3^) = Area (m^2^) × Depth (m) (3)

#### 2.4.3. Determination of Nutritional Content (NC)

To preserve nutrients and avoid further metabolic reactions, grain was freeze-dried using a model RV3 vacuum freeze drier (Edwards, Hampton, NH, USA) after yield determination. Thereafter, samples were ground using a coffee grinder (Mellerware, Durban, South Africa) and sent to the KZN Department of Agriculture and Rural Development Plant Nutrition Lab. The nutrients analysed per dry matter basis included macro-nutrients (fat and protein) and micro-nutrients [calcium (Ca), zinc (Zn), iron (Fe)].

Determination of macro nutrients (fat and protein) followed the Association of Official Analytical Chemists (AOAC) standard procedures for nutrient analysis [34]. Dry matter was determined by drying samples in a fanned oven at 100 °C for 24 h. Nitrogen (N) was determined by the micro-Kjeldahl method. Thereafter, crude protein was calculated as:

N × 6.25(4)

Crude fat was determined according to the soxhlett procedure. Ash was determined by igniting fibre samples in a furnace at 550 °C overnight. The carbohydrate content was then determined as the difference between 100% and addition of the percentages of moisture, fat, crude protein, and crude fibre. The mineral composition (Ca, Zn, Fe) were determined using the dry ashing (DA) technique [34]. An aliquot of 25 mL was placed in crucibles. Thereafter, samples were placed in an oven set at 50 °C to heat overnight. Following this, crucibles with residues obtained after vaporisation of water and most organic compounds were introduced in a high temperature muffle furnace and ashed at 450 °C for 24 h. Thereafter, samples were cooled and residues treated with nitric acid while on warm hot plate. Samples were then transferred back to the muffle furnace for 24 h. White ashes obtained were dissolved in a beaker with 20 mL 5% (v/v) nitric acid. The solution was then transferred to a 25 mL volumetric flask by rinsing with 5% v/v nitric acid. The solution then was used to determine Ca, Zn, Fe using an atomic absorption spectrophotometer (AAS) (Analytikjena AG, Jena, Germany).

#### 2.4.4. Determination of Nutritional Water Productivity (NWP)

Nutritional water productivity was calculated based on the formula by Renault and Wallender [4]:

NWP = (Ya/ET) × NC(5)

where NWP is the nutritional water productivity (nutrition m^−3^ of water evapotranspired), Ya is the actual harvested grain yield (kg·ha^−1^), ET is the actual evapotranspiration (m^3^·ha^−1^), and NC is the nutritional content per kg of product (nutrition unit·kg^−1^).

### 2.5. Data Analysis

Several factors affected the final data collection. In particular, data for cowpea were missing at Ukulinga due to animal attacks, hence no cowpea data are reported for both 2015/16 and 2016/17 season. At Umbumbulu, there was a hailstorm during 2015/16 which damaged plants. This occurred after the planting window and experiments could not be replanted, hence no data are reported for Umbumbulu during 2015/16. These considerations were taken into account as part of data analyses. Data from Ukulinga (the irrigation treatments) and from the three sites (rainfed trials) were analysed separately. For both data sets, data of the two seasons (2015/16 and 2016/17) were subjected to Bartlett’s test for homogeneity of variance in GenStat^®^ 18th Edition (VSN International, London, UK). Results of both data sets showed evidence of non-homogeneity between the two seasons hence a separate analysis of the seasons was conducted. The data sets (the irrigation treatments) and (the three sites) were subjected to analysis of variances (ANOVA) using GenStat^®^ version 18 (VSN International, London, UK). Least significance difference (LSD) was used to separate means at the 5% level of significance.

## 3. Results

### 3.1. Rainfall

**Figure 2:**
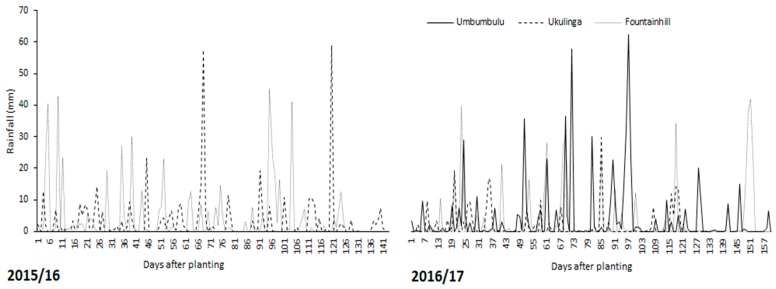
Rainfall (mm) observed at three sites (Ukulinga Research Farm, Umbumbulu Rural District and Fountainhill Estate) during 2015/16 and 2016/17 season.

Total rainfall at Ukulinga and Fountainhill during 2015/16 was 445 and 583 mm, respectively. During 2016/17, total rainfall observed at Ukulinga, Fountainhill and Umbumbulu was 235, 395 and 595 mm, respectively. At Ukulinga during 2015/16, ≈25% of the total rainfall (120 mm) was received in two rainfall events [68 and 120 days after planting (DAP)] (Figure 2). During 2015/16, daily rainfall at Fountainhill did not exceed 45 mm and it was observed that ≈20% of the total rainfall was received during the first 14 days while ≈25% was received between 95 and 106 DAP. At Ukulinga, during 2016/17, rainfall did not exceed 30 mm for all the rain days. In addition to being low (235 mm), rainfall was also sparsely distributed (Figure 2). At Umbumbulu, where the highest rainfall was observed during 2016/17 (595 mm), it was observed that 120 mm of this rainfall was received in two days (72 and 97 DAP).

### 3.2. Nutritional Content in Response to Water Regimes

With respect to fat content, it was observed that groundnut had the highest fat content across all seasons and water treatments (Table 2). For fat content, groundnut had >900% more than the other crops, across all seasons and water treatments. During 2016/17, bambara groundnut fat content was as low as 6 g·kg^−1^. For all the crops, there was no discernible pattern with respect to the water treatment (Table 2). However, during 2015/16, groundnut fat content under the RF treatment was ≈100 g·kg^−1^ less than under OI and DI. Groundnut had more protein content during 2015/16, though the differences were not as high as for fat content. Bambara groundnut had the lowest protein content (200–258 g·kg^−1^) (Table 2). The highest difference between protein of groundnut and dry bean was 14%. This was observed under RF conditions. During 2016/17 dry bean had the highest protein under RF conditions (287 g·kg^−1^), and the lowest protein under DI (247 g·kg^−1^) (Table 2).

For the micronutrients, dry bean had the highest Ca content during 2015/16 under all the water treatments. Under rainfed conditions, Ca content in dry bean was ≈100% more than groundnut and bambara groundnut. During 2016/17, bambara groundnut showed high Ca content under DF conditions (100% more than dry bean) (Table 2). Contrary to the macronutrients, groundnut was inferior to dry bean and bambara groundnut, showing the lowest Ca content (100 mg·kg^−1^). For Zn and Fe content there was no clear pattern between the crops and the water treatments. For Zn content, the differences between the crops ranged between (5–15% which was lower compared to the differences observed for fat content (22–900%). For Fe content, it was observed that during 2015/16, dry bean had 200–350% more Fe content compared to bambara groundnut and groundnut under all the water treatments. Groundnut had the lowest Fe (Table 2). During 2016/17, it was interesting to observe that under OI, bambara groundnut had the highest Fe content (84.1 mg·kg^−1^), while groundnut had the highest Fe content under DI (102.9 mg·kg^−1^) and dry bean had the highest Fe under RF (104.6 mg·kg^−1^) (Table 2).

### 3.3. Nutritional Content in Response to Environments

Across environments, groundnut maintained its superiority with respect to fat content. Groundnut maintained a high fat content of >900% compared to the other crops. The lowest fat content (4.87 g·kg^−1^) was observed for cowpea at Fountainhill during 2016/17. Under the irrigation treatments, there was no discernible pattern of crop performance with respect to protein content. Across environments however, groundnut had the highest protein content during both seasons 275–325 g·kg^−1^). It was also observed that bambara groundnut had the lowest protein content across environments (205–253 g·kg^−1^). During 2016/17, for all the crops, the lowest protein content was observed at Ukulinga (205–275 g·kg^−1^) relative to Fountainhill (214–325 g·kg^−1^) and Umbumbulu (225–316 g·kg^−1^) (Table 3).

Under the irrigation regimes, high Ca content in dry bean was limited to 2015/16 (Table 3). Under different environments, dry bean had the highest Ca content during both seasons (1.24–1.54 mg·kg^−1^) (Table 3). At Fountainhill and Umbumbulu, cowpea, had the 2nd highest Ca content (740–1370 mg·kg^−1^) after dry bean. Groundnut, had the highest fat and protein but had the lowest Ca content at Ukulinga and Fountainhill during both seasons (<550 mg·kg^−1^). Similar to irrigation treatments, there was no clear pattern on crop performance with respect to Zn content across environments (Table 3). However, it was observed that during both seasons, cowpea had the highest Zn content at Fountainhill (67.8 and 53.8 mg·kg^−1^). It was also observed that at all sites during 2015/16 and at Umbumbulu and Fountainhill during 2016/17, bambara groundnut had the lowest Zn (<33.2 mg·kg^−1^). For bambara groundnut and cowpea, there was a Zn content difference of ≈100%, with cowpea having the highest (Table 3). For Fe content, dry bean and cowpea had the highest Fe content (61.6–104.6 mg·kg^−1^). Fe in groundnut and bambara groundnut, ranged between 21.3 and 63.8 mg·kg^−1^, 100–300% lower than dry bean and cowpea (Table 3). Comparing the environments, it was observed that all the crops had the highest Fe (42.4–104.6 mg·kg^−1^) at Ukulinga during 2016/17. This was the environment where all the lowest protein for all the crops was observed (Table 3).

### 3.4. Nutritional Water Productivity in Response to Water Regimes

During 2015/16, results of yield and NWP for all the nutrients (protein, fat, Ca, Zn and Fe) showed significant differences (*p* < 0.05) among the crops. Water treatments were not significantly different (*p* > 0.05) (Table 4). The interaction between water treatments and crops was significantly different (*p* < 0.05) for grain yield, NWP_fat_ and NWP_protein_. Under OI, the highest yield was observed for dry bean (2260 kg·ha^−1^). Dry bean also had the lowest ET (2680 m^−3^) translating to high productivity (Table 4). This resulted in the highest NWP_protein_ (220 g·m^−3^), despite the crop not having the highest protein content under OI. The high Ca (1270 mg·kg^−1^) and Fe content (85 mg·kg^−1^) observed for dry bean under OI translated to high NWP_Ca_ (1060 mg·m^−3^) and NWP_Fe_ (71.9 mg·m^−3^). Groundnut had high fat content resulting in the highest NWP_fat_ (249 g·m^−3^). For bambara groundnut, low NWP for all the nutrients was as a result of combined effect of low yield, high ET and low nutritional content (Table 4).

In addition to the high fat and protein content observed for groundnut under DI, it had the highest yield (200% more than the other crops) (Table 5). This resulted in higher NWP_fat and protein_ (4956 kcal·m^−3^, 406 g·m^−3^, 314 g·m^−3^) under DI. It was interesting to observe that despite groundnut having the lowest Ca and Fe, it had the second highest NWP_Ca and Fe_, (590 and 35.1 mg·m^−3^, respectively) because of the high grain yield (2900 kg·ha^−1^) (Table 5). For bambara groundnut, results were consistent to the OI treatment—it had the lowest NWP for all the nutrients. Dry bean had the highest NWP_Ca and Fe_ (>300% more than groundnut and bambara groundnut) (Table 4).

During 2016/17, results of grain yield and NWP were similar to 2015/16—significantly different among crops (*p* < 0.05) and not significantly different among irrigation treatments (*p* > 0.05) (Table 5). The interaction between crops and water regime was only significant for NWP_fat, Ca and Fe_. During 2016/17, dry bean had the highest grain yield (1081–1296 kg·ha^−1^) and lowest ET (1430–1950 m^−3^) across all water treatments. As a result, the highest NWP_protein, Ca, Zn and Fe_ was highest for dry bean across water treatments. Although groundnut had 800% more fat under DI, dry bean had a higher NWP_fat_ (42 g·m^−3^) due to the high grain yield and low ET. During 2015/16, groundnut performed better than bambara groundnut. In 2016/17 due to low grain yield for bambara groundnut and groundnut, the crops had similar NWP_protein, Ca, Zn and Fe_ despite groundnut having higher nutrient content than bambara groundnut (Table 2 and Table 5).

### 3.5. Nutritional Water Productivity in Response to Environments

During 2015/16, sites were not significantly different for grain yield (*p* > 0.05) while NWP for all the nutrients (protein, fat, Ca, Zn and Fe) was significantly different (*p* < 0.05). Grain yield and NWP for all the nutrients (protein, fat, Ca, Zn and Fe) were significantly different (*p* < 0.05) among the crops (Table 6). The interaction between crop and site was significant (*p* < 0.05) for grain yield and NWP for all the nutrients (protein, fat, Ca, Zn and Fe). At Fountainhill, despite bambara groundnut having the highest yield (1978 kg·ha^−1^), it did not have the highest NWP for all the nutrients because of high ET (4370 m^3^) and low nutritional content (Table 3 and 6). Groundnut had the highest macro nutrient content (Table 2 and Table 3) which was translated to the highest NWP_fat and protein_ (2575 kcal·m^−3^, 197 g·m^−3^, 148 g·m^−3^, respectively). Dry bean had the highest NWP_Fe and Ca_ (>39.7 mg·m^−3^ and >570 mg·m^−3^). Despite low grain yield of cowpea, it had the highest NWP_Zn_ (26.3 mg·m^−3^) due to the high Zn content (67.8·mg·kg^−1^). Comparing the two sites, it was observed that Ukulinga yielded better (1950 kg·ha^−1^ and had lower ET (2660 m^3^) than Fountainhill (1560 kg·ha^−1^ and 3547 m^3^, respectively. This led to 60–110% higher NWP for all the nutrients (protein, fat, Ca, Zn and Fe) at Ukulinga compared to Fountainhill. 

During 2016/17, results of crops were significantly different (*p* < 0.05) for NWP_fat, Ca, Zn and Fe_. For sites, NWP_protein, Ca, Zn and Fe_ were significantly different (*p* < 0.05). The interaction between crop and site was significantly different (*p* < 0.05) for NWP_fat, protein and Zn_ (Table 7). During 2015/16, it was observed that Ukulinga was better performing than Fountainhill. In 2016/17, Fountainhill was the best performing site. At Fountainhill, grain yield, NWP_fat, protein, Ca and Zn_ was ≈100% more than at Umbumbulu and Ukulinga. Groundnut had the highest NWP_fat and protein_ at Fountainhill and Umbumbulu (Table 7). At Ukulinga, dry bean grain yield was high, and ET was low, contributing to the highest NWP_protein_ (2347 kcal·m^−3^ and 204 g·m^−3^, respectively). Similar to results of 2015/16, dry bean had the highest NWP_Fe_ at Ukulinga and Fountainhill (79 and 46.6 mg·m^−3^), however due to the low grain yield at Umbumbulu (282 kg·ha^−1^), the crop did not have the highest NWP_Fe_ (9.1 mg·m^−3^). 

## 4. Discussion

The objectives of the study were to determine the nutrient content and NWP of selected indigenous and major grain legumes in response to water regimes and production environments. To the best of our knowledge, this is the first study providing a comparative study of nutritional content and NWP of indigenous and major grain legumes grown under the same conditions. Previous studies that have compared nutritional content and NWP of grain legumes have relied on information obtained from a range of studies that were conducted under different environmental conditions [4,35].

Crops differed in their nutritional content. Groundnut had higher fat content relative to the other crops; a 100 g serving of groundnut can supply the Recommended Dietary Allowance (RDA) of fat (40–78 g). A gram of fat contains ≈37.6 kJ of energy, hence fat rich foods are good sources of energy. The high fat content of groundnut has been explored through processing into peanut butter and extraction of oil for household use. This makes groundnut a multi-purpose grain legume, and partly explains the reason why groundnut is an important and major grain legume. However, over consumption of groundnut poses risk associated with excess fat consumption, which is one of the major causes of obesity [36,37]. In semi- and arid regions, 30% of the population is overweight and obese [1], hence the promotion of groundnut needs to be accompanied with proper consumption recommendations. This also supports the need to diversify grain legumes to avoid over reliance on a few major legumes such as soybean and groundnut that have high fat content.

For all the grain legumes, protein content was between 205 and 325 g·kg^−1^, implying that a 100 g portion of legume supplies 40–60% of protein RDA (50 g). This confirms arguments that legumes can be promoted as alternatives to meat, to avoid protein energy malnutrition [22,23]. Legumes have also been associated with containing appreciable amounts of micronutrients [38,39,40]. In the semi- and arid regions, Fe, Ca and Zn are among the problematic micronutrients as their deficiency has devastating consequences such as anaemia in women of reproductive age and birth defects in children [37]. For Fe, Ca, Zn, the RDA for an adult is 18 mg, 1000 mg and 11 mg, respectively [41]. Fruits and vegetables are the major sources of micronutrients, but they are not always available due to price and seasonality. Dry bean and cowpea have the potential to supply 40 to 60% of Fe and Zn RDA. In the case of Zn, this study showed that cowpea and dry bean contained ≈500% more Zn than leafy vegetables that have been observed to contain 2.9 to 15.1 mg·kg^−1^ [24]. While vegetables such as spider flower contain more Fe than grain legumes (200 mg·kg^−1^), Fe content of grain legumes is comparable to those observed for vegetables such as Swiss chard and cabbage (38.80–98.40 mg·kg^−1^) [24]. This study brings a new perspective that vegetables are not the only major source of micronutrients but legumes’ micronutrient value is comparable to that of leafy vegetables. This supports the role of legumes in increasing dietary diversity as they can complement cereals and vegetables in diets to meet the required nutrients for a healthy life [23].

Among the grain legumes under study, bambara groundnut had the lowest macro- and micro nutrient content. Nutrient content of bambara groundnut observed in this study were in the same range of those observed in other studies [42,43,44]. Amarteifio et al. [43] assessed micronutrient content of various landraces from Botswana, Namibia and Swaziland. They observed large variability within landraces and interestingly landraces from Swaziland had higher micronutrient content than landraces from Namibia and Botswana. This demonstrates that some bambara groundnut landraces are more nutrient dense than others. Findings of this study are a first, as they suggest that non - uniformity in nutrient content of bambara groundnut is not limited to different landraces but may also occur within the same landrace. During 2016/17, bambara groundnut had ≈100% more Ca under DI compared to the other treatments. This non-uniformity in nutrient content within and across bambara groundnut landraces may hamper its promotion in the semi- and arid tropics. This calls for breeding efforts to select for nutrient dense landraces that can be used in breeding for high and uniform nutrient content.

Nutrient content of crops differed across water treatments and environments. When rainfall was low (Ukulinga during 2016/17), protein content for all the crops was also low. The low protein content under water limited conditions is attributed to low nitrogen (N) uptake by the plant. Nitrogen is correlated to protein content because it is important for synthesis of amino acids which are building blocks of proteins. Under water limited conditions, the activity of the enzyme that converts nitrogen to a form that is readily available to plants (nitrate reductase) is reduced [45]. This ultimately reduced N availability to the plant [45], and consequently protein synthesis was reduced. This implies that water stress does not only affect yield, but can also affect protein content of crops. Fe content was higher at Ukulinga compared to the other sites. Fe is not readily mobile to different plant organs and its delivery to seeds depends on a continuous Fe transport system [45,46].The moisture of soil affects Fe availability. Wet soils have greater Fe availability for plants due to higher Fe^2+^/Fe^3+^ ratio [45,46]. Ukulinga was characterised by shallow soil profile and clay soil hence good water holding capacity. This could have enhanced Fe mobility from roots to seeds. Inherent environmental conditions influenced grain nutrient content but there is still a dearth of information on how inherent environmental conditions and plant nutrient availability affects grain nutrient content in different crops.

To the best of our knowledge, this is the first study to determine the NWP of grain legumes based on in situ measurements and not estimates, hence results are more reliable. Nutritional water productivity varied significantly among the crops. With respect to fat productivity, groundnut was the most productive producing up to 400 g·m^−3^, respectively. This was because of high fat content. For NWP_Fe, Zn and Ca_, dry bean was the most productive followed by cowpea. For groundnut, despite the high grain yield, NWP_Fe, Zn and Ca_ was low due to poor nutrient content. This highlights the need for crop diversification to maximise nutritional productivity as crops showed different qualities. Fe, Zn and Ca contents of dry bean and cowpea observed in this study were comparable to those observed for leafy vegetables. However, NWP_Fe, Zn and Ca_ observed for leafy vegetables by Nyathi et al. [24] were higher (≈200%) than those observed by this study for grain legumes. This could be because leafy vegetables relatively used less water (1210–3260 m^−3^) and had higher yield (600–9500 kg·ha^−1^) than the grain legumes under study. For maximum benefit of Fe, Zn and Ca under water limited conditions, vegetables would be the recommended option as they are more productive. This highlights the importance of merging aspects of water use, yield and nutritional content for effective recommendations on tackling food and nutritional security. 

The major legumes (groundnut and dry bean), had the highest protein water productivity, relative to the indigenous grain legumes. In the case of groundnut, it was mostly as a result of high protein content and high yield observed for the crop. For dry bean, high protein water productivity was as a result of low ET and high protein content. For the indigenous grain legumes (cowpea and bambara groundnut), protein water productivity was low due to low protein content, high ET and low grain yield for bambara groundnut and low yield for cowpea. If indigenous grain legumes are to be promoted for crop diversification, there is need for yield and nutritional content improvements, to improve protein water productivity. When comparing protein water productivity values of grain legumes (100–300 g·m^−3^) to that estimated for meat products (12–60 g·m^−3^) [35], it is interesting to note that despite meat being the highest protein source, legumes are more productive. This is because water consumption in legume production is less than water consumption for production of meat. This further supports the promotion of legumes as protein alternatives in water scarce areas as they relatively use less water compared to production of meat [35]. 

Environments had a significant effect on NWP. This was mostly as a result of yield instability across environments. Fluctuations in NWP followed fluctuations in grain yield. Low grain yield caused low NWP. There has been emphasis on improving yield stability in the context of food security. This study highlights a new insight that yield stability also affects NWP and improving yield stability not only ensures continuous availability of grain but also ensures continuous nutritional gain. Water regimes did not have a significant effect on NWP. Grain yield was also not significantly affected by water regimes. This implies that there is scope to tackle the challenge of food and nutritional security in the semi- and arid tropics under rainfed conditions. 

## 5. Conclusions

Groundnut had a higher fat content relative to the other crops. Dry bean and cowpea had the highest micronutrient and have potential to supply 40 to 60% of Fe and Zn RDA. This highlighted their potential in increasing dietary diversity as they can serve as complements to cereals and vegetables in diets to meet the required nutrients for a healthy life. The protein content of all the grain legumes showed potential to supply 40–60% of protein RDA. This confirmed the role of legumes as a source of dietary protein among poor rural people who may not be able to afford meat and dairy products. Bambara groundnut had the lowest macro- and micro nutrient content. In addition to the non-uniformity in nutrient content of different bambara groundnut landraces, this study was a first to observe non-uniformity in nutrient content within the same landrace. This calls for breeding efforts to breed for nutrient density and uniformity in bambara groundnut. Protein content reduced when rainfall was low. Fe content was higher under clay soil. This highlights that climate and edaphic conditions do not only affect yield but nutritional content also. The major legumes (groundnut and dry bean), had the highest protein water productivity, relative to the indigenous grain legumes. For NWP_Fe, Zn and Ca_, dry bean and cowpea were more productive. Environments had a significant effect on NWP, hence the hypothesis was rejected. Differences in NWP across environments were due to yield instability across environments. Yield stability of grain legumes is key to tackling food and nutrition insecurity. In the case of water regimes, the hypothesis could not be rejected as water regimes did not significantly affect NWP. This implies that there is scope to tackle the challenge of food and nutrition security in the semi- and arid tropics under rainfed conditions. While the results of the current study may be preliminary, they provide useful initial insights on how increasing food production and crop diversity can be linked to addressing nutritional outcomes. This study only provides a first insight about the nutrient content and nutritional water productivity of a limited number of selected grain legumes in response to the production environment. This first study therefore requires detailed follow-up studies to also include cowpea data. In addition, such future studies should provide more experimental data and explore effects of additional factors such as management practices (fertiliser levels and plant density), climate and edaphic factors on nutrient content and NWP for a range of legumes.

## Figures and Tables

**Table 1:** Site characteristics of the three selected sites (Ukulinga Research Farm, Umbumbulu Rural District and Fountainhill Estate).

Site	Ukulinga Research Farm	Umbumbulu Rural District	Fountainhill Estate
Coordinates	29°37′S; 30°16′E	29°98′S; 30°70′E	29°44′S; 30°54′E
Altitude (m.a.s.l.)	750	593	1020
Annual rainfall	694	1200	905
Average temperature	25	28	20.4
Average max temperatures	26	27	29
Average min temperatures	10	13	17
Soil type	Heavy Clay	Clay-Loam	Sandy
Bio-resource group	Moist Coast Hinterland Ngongoni Veld	Moist Coast Forest, Thorn and Palm Veld (Moist Coast)	Moist Midland Mistbelt

**Table 2:** Macro (protein and fat) and micro (Ca, Zn and Fe) nutrients of four grain legume crops (groundnut, bambara groundnut, dry bean and cowpea) grown under varying irrigation regimes (optimum irrigation, deficit irrigation and rainfed) over two seasons (2015/16 and 2016/17).

			Fat	Protein	Ca	Zn	Fe
			g·kg^−1^		mg·kg^−1^		
2015/16	OI	Groundnut	406.65	290.16	710	44.43	38.00
		Bambara	10.24	210.55	670	28.27	39.01
		Dry Bean	50.27	260.18	1270	30.67	85.04
	DI	Groundnut	400.04	310.58	600	37.31	35.02
		Bambara	40.06	200.82	630	32.82	39.03
		Dry Bean	40.36	300.89	990	44.03	103.04
	RF	Groundnut	301.19	310.19	550	37.12	30.09
		Bambara	10.27	230.87	590	33.23	42.00
		Dry Bean	40.60	270.32	1400	33.95	87.00
2016/17	OI	Groundnut	405.44	249.77	860	32.92	47.90
		Bambara	57.24	231.13	580	30.36	84.17
		Dry Bean	10.13	287.77	1170	33.28	69.60
	DI	Groundnut	418.50	288.82	1110	32.79	102.96
		Bambara	6.21	258.88	1260	32.59	60.75
		Dry Bean	62.99	247.72	650	25.07	70.01
	RF	Groundnut	438.79	275.59	100	35.70	63.84
		Bambara	59.57	205.55	600	29.47	42.47
		Dry Bean	17.90	270.03	1140	29.39	104.64

**Table 3:** Macro (protein and fat) and micro (Ca, Zn and Fe) nutrients of four grain legume crops (groundnut, bambara groundnut, dry bean and cowpea) grown at three different sites (Fountainhill Estate, Ukulinga Research Farm and Umbumbulu Rural District) over two seasons (2015/16 and 2016/17).

			Fat	Protein	Ca	Zn	Fe
			g·kg^−1^		mg·kg^−1^		
2015/16	Ukulinga	Groundnut	300.19	310.19	550	37.23	30.93
		Bambara groundnut	10.27	230.87	590	33.31	42.09
		Dry Bean	40.60	270.32	1400	33.59	87.02
	Fountainhill	Groundnut	430.15	325.87	310	45.86	29.64
		Bambara groundnut	40.36	214.54	460	30.95	28.03
		Dry Bean	14.32	282.61	1240	42.52	85.04
		Cowpea	47.13	272.99	740	67.38	96.86
2016/17	Ukulinga	Groundnut	438.79	275.59	100	35.02	63.46
		Bambara groundnut	59.57	205.55	600	29.71	42.72
		Dry Bean	17.90	270.03	1140	29.94	10.42
	Fountainhill	Groundnut	470.29	324.42	330	46.49	21.75
		Bambara groundnut	47.42	253.20	620	28.86	23.98
		Dry Bean	14.26	277.82	1540	42.28	76.46
		Cowpea	4.87	314.06	1160	51.76	60.84
	Umbumbulu	Groundnut	448.75	316.12	510	41.61	26.91
		Bambara groundnut	61.74	225.55	380	27.05	21.24
		Dry Bean	22.91	303.86	1430	42.23	67.96
		Cowpea	12.09	295.92	1370	40.20	61.04

**Table 4:** Yield, Evapotranspiration (ET) and nutritional water productivity (NWP) (protein, fat, Ca, Zn, and Fe), of three legume crops (dry bean, groundnut and bambara groundnut) grown under three water treatments (OI, DI and RF) during the 2015/16 season.

Water Treatments	Crop Species	Grain Yield	ET	NWP_fat_	NWP_protein_	NWP_Ca_	NWP_Zn_	NWP_Fe_
		kg·ha^−1^	m^−3^	g·m^−3^		mg·m^−3^		
OI	Dry bean	2260a	2680	44.00c	220.30b	1060a	25.80	71.90a
	Groundnut	1950ab	3160	249.20b	178.80c	440b	27.20	23.30b
	Bambara groundnut	1480b	3170	5.80c	100.70d	310c	13.20	18.30b
DI	Dry bean	1400b	2390	27.30	193.30b	620b	27.50	64.70a
	Groundnut	2900a	2920	406.00a	314.70a	590b	37.20	35.10b
	Bambara groundnut	1410b	2630	21.80c	111.60d	340c	17.60	21.10b
RF	Dry bean	1960a	2380	38.00c	225.40b	1150a	28.00	71.80a
	Groundnut	2770a	2830	308.20b	305.60a	450b	36.40	30.30b
	Bambara groundnut	1090b	2770	5.00c	94.40d	230c	13.10	16.70b
Significance (*p* = 0.05)	Crops	<0.001		<0.001	<0.001	<0.001	<0.001	<0.001
	Water regime	* ns		* ns	* ns	* ns	* ns	* ns
	Crops * Water regime	0.031		0.028	0.040	* ns	* ns	* ns
	LSD _(*p* = 0.05)_	1069		78.00	32.20	410		26.63

* ns: Not significant at *p* = 0.05.

**Table 5:** Yield, water use and NWP (protein, fat, Ca, Zn, and Fe), of three legume crops (dry bean, groundnut and bambara groundnut) grown under three water treatments (OI, DI and RF) during the 2016/17 season.

Water Treatments	Crop Species	Grain Yield	ET	NWP_fat_	NWP_protein_	NWP_Ca_	NWP_Zn_	NWP_Fe_
		kg·ha^−1^	m^−3^	g·m^−3^		mg·m^−3^		
OI	Dry bean	1296a	1950	6.70d	191.00a	1140a	22.90a	81.20a
	Groundnut	585b	3450	68.60a	42.30b	140b	5.57b	46.20b
	Bambara groundnut	466b	3060	8.70d	35.10b	80b	4.61b	12.80c
DI	Dry bean	1098a	1630	42.40b	166.30a	430b	16.86a	47.10b
	Groundnut	362b	2800	34.70c	23.90b	90b	2.72b	8.50c
	Bambara groundnut	402b	2560	1.10e	45.00b	220b	5.67b	10.60c
RF	Dry bean	1081a	1430	13.50d	204.00a	1110a	22.18a	79.00a
	Groundnut	267b	2490	46.90b	29.50b	100b	3.82b	6.80c
	Bambara groundnut	292b	2320	7.50d	25.90b	80b	3.71b	5.30c
Significance (*p* = 0.05)	Crops	<0.001		<0.001	<0.001	<0.001	<0.001	<0.001
	Water regime	* ns		* ns	* ns	* ns	* ns	* ns
	Crops * Water regime	* ns		<0.001	* ns	0.022	* ns	<0.001
	LSD _(*p* = 0.05)_	538.5		11.17	72.30	380	8.30	24.42

* ns: Not significant at *p* = 0.05.

**Table 6:** Yield, ET and NWP (protein, fat, Ca, Zn, and Fe), of four legume crops (dry bean, cowpea, groundnut and bambara groundnut) grown at two sites (Fountainhill Estate and Ukulinga Research Farm) during 2015/16 season.

Water Treatments	Crop Species	Grain Yield	ET	NWP_fat_	NWP_protein_	NWP_Ca_	NWP_Zn_	NWP_Fe_
		kg·ha^−1^	m^−3^	g·m^−3^		mg·m^−3^		
Fountainhill	Dry bean	1456ab	3130	6.64c	131c	570b	19.87b	39.73b
	Groundnut	1594ab	3490	197.05b	148.8c	140c	21.00b	13.36c
	Bambara groundnut	1978a	4370	18.26c	97.1c	200c	13.89c	12.90c
	Cowpea	1214b	3200	18.28c	105.8c	280c	26.30b	37.33b
Ukulinga	Dry bean	1960a	2380	38.00c	225.40b	1150a	28.00a	71.80a
	Groundnut	2770a	2830	308.20a	305.60a	450b	36.40a	30.30b
	Bambara groundnut	1090b	2770	5.00c	94.40c	230c	13.10c	16.70c
Significance (*p* = 0.05)	Crops	0.032		<0.001	<0.001	<0.001	0.001	<0.001
	Site	* ns		0.003	<0.001	<0.001	0.007	<0.001
	Crops * Site	0.003		0.002	0.007	0.002	0.046	0.015
	LSD _(*p* = 0.05)_	745.9		44.38	63.27	180	8.76	12.48

* ns: Not significant at *p* = 0.05.

**Table 7:** Yield, water use and NWP (protein, fat, Ca, Zn, and Fe), of four legume crops (dry bean, groundnut and bambara groundnut) grown under three water treatments (Fountainhill Estate, Ukulinga Research Farm and Umbumbulu Rural District) during 2016/17 season.

Site	Crop Species	Grain Yield	ET	NWP_fat_	NWP_protein_	NWP_Ca_	NWP_Zn_	NWP_Fe_
		kg·ha^−1^	m^−3^	g·m^−3^		mg·m^−3^		
Fountainhill	Dry bean	1302a	2140	8.67c	169a	930a	25.80	46.67b
	Groundnut	2387a	2870	390.8a	269.6a	270b	38.61	18.09c
	Bambara groundnut	1359a	2650	24.31c	129.8	310b	14.86	12.25c
	Cowpea	1011a	2730	1.80c	116.3b	420b	19.16	22.32c
Umbumbulu	Dry bean	282c	2080	3.10d	41.2b	190b	5.96	9.12c
	Groundnut	1213a	2340	231.91b	163.4a	260b	21.43	13.96c
	Bambara groundnut	725b	2840	15.6c	57.6b	90b	7.1	5.44c
	Cowpea	953ab	3340	1.80c	84.4b	390b	11.56	17.58c
Ukulinga	Dry bean	1081a	1430	13.50c	204.00a	1110a	22.18	79.00a
	Groundnut	267b	2490	46.90c	29.50b	10c	3.82	6.80c
	Bambara groundnut	292b	2320	7.50c	25.90b	80b	3.71	5.30c
Significance (*p* = 0.05)	Crops	* ns		<0.001	* ns	0.008	0.027	0.006
	Site	0.002		* ns	0.010	0.012	0.002	0.010
	Crops * Site	* ns		<0.001	0.004	* ns	0.007	* ns
	LSD _(*p* = 0.05)_	1007.3		91.89	113.5	350		17.33

* ns: Not significant at *p* = 0.05.
